# Emergence of Rickettsial Infection in Rainbow Trout (*Oncorhynchus mykiss*) Fry Displaying the Appearance of Red Mark Syndrome in Korea

**DOI:** 10.3390/microorganisms7090302

**Published:** 2019-08-29

**Authors:** Woo Taek Oh, Sib Sankar Giri, Saekil Yun, Hyoun Joong Kim, Sang Guen Kim, Sang Wha Kim, Jeong Woo Kang, Se Jin Han, Jun Kwon, Jin Woo Jun, Se Chang Park

**Affiliations:** 1 Laboratory of Aquatic Biomedicine, College of Veterinary Medicine and Research Institute for Veterinary Science, Seoul National University, Seoul 08826, Korea; 2 Department of Aquaculture, Korea National College of Agriculture and Fisheries, Jeonju 54874, Korea

**Keywords:** *Oncorhynchus mykiss*, red mark syndrome (RMS), Rickettsial infection

## Abstract

Red mark syndrome (RMS) is a fish disease caused by the infection of Rickettsial agents, especially affecting rainbow trout (*Oncorhynchus mykiss)*. The disease is prevalent in many countries in Europe (France, Switzerland, Italy, and Slovenia), South America (Chile), North America (USA), and even Asia (Japan). However, it has not been reported in Korean aquaculture. In February 2019, rainbow trout presenting red spot lesions with swollen features on the lateral side of their body were observed at a hatchery in Korea. Fishes showing those clinical signs were fry weighing 25 ± 5 g. Moreover, the fish showing the red spot lesions were found dead, which suggests an outbreak of a mortality-causing disease. The symptoms were similar to those of RMS, and we identified the presence of *Rickettsia*-like organisms associated with this disease using polymerase chain reaction (PCR), sequencing, histopathologic examination, and transmission electron microscopy. The distinct features of this infection, compared to that in previous reports, were that RMS occurred in small-sized fish and accompanied mortality. Additionally, the presence of the *Rickettsia* agent was accompanied with outbreak of the disease. Therefore, this is the first report of RMS outbreak in rainbow trout fisheries in Korea.

## 1. Introduction

Red mark syndrome (RMS) is a skin condition related to *Rickettsia*-like organism (RLO) infection that can develop in rainbow trout under specific circumstances [1]. RMS is only observed in fish at a water temperature below 15 °C, from which another name for the disease was derived: cold water strawberry disease [2]. Often called strawberry disease, RMS is known as a non-fatal disease appearing usually during the winter and spring [3]. In the USA, a disease with similar clinical signs is observed, but in different water conditions. Thus, the disease was named as warm water strawberry disease and rash [4]. RMS first appeared in a single rainbow trout farm located in Scotland, and then spread simultaneously throughout the rest of the United Kingdom [5]. Furthermore, the disease spread sporadically to many countries, including continental European countries, the USA, Chile, and Japan, which caused many problems [6]. The affected fish are mainly market-sized fish weighing more than 500 g. Therefore, economic losses occur because the clinical signs of RMS lead consumers to think that the fish are inappropriate for consumption [7]. Fish with RMS are generally known not to display loss of appetite or inhibited growth [8]. Moreover, despite causing high morbidity at around 5% to 50%, this disease is known not to be associated with fish mortality [9]. The main causes of the disease remain unclear, but two main pathogens have been studied to elucidate the etiology of RMS, namely RLOs and *Flavobacterium psychrophilum* [10]. A phylogenetic study proved that the RLOs associated with RMS belong to the recently described Midichloriaceae family and order Rickettsiales [6]. However, studies reported that *F. psychrophilum* was not isolated or detected in RMS-affected tissues [10]. Immunohistochemistry, transmission electron microscopy (TEM), PCR of the 16s rRNA gene, and histopathology analyses can be conducted for diagnosis of this disease. In recent studies, intracytoplasmic oval-shaped microorganisms were detected within macrophages and erythrocytes in the skin of RMS-affected fish, which indicates that these microorganisms are a possible cause of this disease [1,11]. The present study is the first report of RMS among a farmed rainbow trout population in Korea. In February 2019, a rainbow trout farm located in Jeonbuk Province asked for disease diagnosis in view of the appearance of red spot lesions on the skin of rainbow trout fry. The affected fish constituted 20% of the group and showed clinical signs resembling RMS. Accordingly, we investigated the cause of the disease, with a focus on RLO detection. Our results suggest that the RMS outbreak had also occurred in Korea.

## 2. Materials and Methods

### 2.1. Sample Collection and Post Mortem Examination

**Figure 1:**
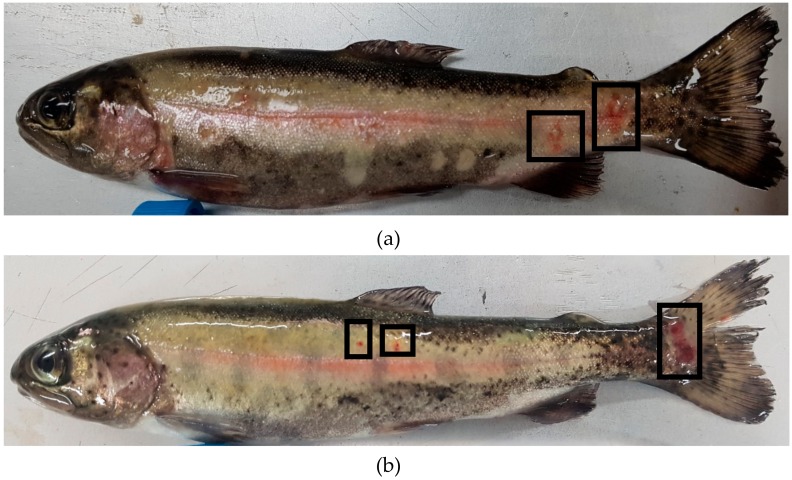
Fish showing clinical signs of red spotted lesions resembling RMS on the lateral side of the body (**a**) and caudal fin (**b**).

The farm was located in Jeonbuk province, Korea. The water was supplemented with ground water in an open circulating water system. Rainbow trout fry were grouped according to their size, and each group was raised in a 35,000 L water tank. The fisheries consisted of four water tanks and approximately 3000 fish were reared on each water tank. Fish with red spot lesions on their skin were collected for disease diagnosis (Figure 1). The size of the affected fish was approximately 25 ± 5 g, and the water temperature was 12 °C. The estimated percentage of the fish showing red mark clinical signs was approximately 20%. The red spot lesions were only observed in rainbow trout fry that weighed less than 30 g.

Fish with clinical signs of red spots (*n* = 5) and fish with no clinical signs but dwelling in the same water tank (*n* = 5) were sent to the laboratory of aquatic biomedicine in Seoul National University for diagnosis. The external clinical signs of the affected fish were evaluated microscopically to detect any bacterial or fungal infection. Because the water temperature was approximately 12 °C and signs of damaged fins were observed in most fish with red mark lesions, it seemed that detection of *F. psychrophilum* was required. For diagnosis of any bacterial infection, the internal organs of fish, including the liver, kidney, and spleen were separated for analysis. The tissues were homogenized in phosphate buffered saline (PBS) and total DNA was extracted using a DNeasy^®^ Blood & Tissue Kit (Qiagen, Valencia, CA, USA), according to the manufacturer’s protocol. Bacterial infection was detected by PCR using specific primers targeting *F. psychrophilum*, *A. salmonicida*, and *P. putida* [12]. To detect viral infection, total RNA was extracted from homogenized tissues using a Patho Gene-spin RNA extraction kit (iNtRON Biotehnology, Daejeon, Korea) and cDNA was synthesized using a PrimeScript 1st strand cDNA Synthesis kit (TaKaRa bio, Shiga, Japan). Viral infection was examined by PCR using specific primers targeting infectious hematopoietic necrosis virus (IHNV), infectious pancreatic necrosis virus (IPNV), and viral hemorrhagic septicemia virus (VHSV) [13,14]. To detect Rickettsial infections, 10 red mark lesions in the affected fish were cut into 1 mm × 1 mm × 1 mm cubes. Five of the cubes were immersed in 2.5% glutaraldehyde solution for use in transmission electron microscopy (TEM), and the others were preserved at −80 °C for use in PCR analysis. To detect *Rickettsia* in internal organs, especially the spleen, the same procedure was performed using separated spleen. For post-mortem examination, the kidney, spleen, and liver of the affected fish were collected. Some of the samples were stored at 4 °C for detection of any bacterial infection and Rickettsial infection, and the rest were immersed in 10% neutralized buffered formalin for histopathological examination. For diagnosis of any other bacterial infection excluding the species detected by PCR, bacteria isolated from organ samples were cultured. The collected organs were homogenized in 300 µL PBS and then incubated on two types of solid agars, which include tryptic soy agar (TSA) and Cytophaga agar, at 18 °C and 25 °C for 48 h.

### 2.2. DNA Extraction and Molecular Detection of RLO

Five RMS lesions were cut into cubes, and the kidney, liver, and spleen of the affected fish were used for DNA extraction. Five fish not showing the signs were also selected and processed by the same procedure for comparison. Next, 50 mg of each sample was homogenized, and total DNA was extracted using a DNeasy^®^ Blood & Tissue Kit (Qiagen, Valencia, CA, USA), following the manufacturer’s protocol. Nested PCR was performed to detect RLO. For the first step of nested PCR, the specific primers RLO1 and RLO2 were used under the same conditions described by Cecchini et al. [15]. For the second step of nested PCR, the specific primers RiFCfw and RiFCrev were used according to the protocol described by Galeotti et al. [16]. The targeted partial 16s rRNA gene of RLO was sequenced and sent for conducting a TA cloning assay. For gene sequence analysis, PCR products were sent to the genomic division of Macrogen (Korea), where nucleotide sequencing was performed using ABI PRISM 3730XL Analyzer with BigDye^®^ Terminator v3.1 Cycle Sequencing Kits (Applied Biosystems, CA, USA).

### 2.3. Phylogenetic Analysis

Sequence of the targeted gene (188 bp) was used for phylogenetic analysis. BLASTn was performed to identify the sequences obtained from the specimen, and the 100 most similar sequences were retrieved for phylogenetic analysis. The sequences were aligned using BioEdit software [17]. The consensus sequences were imported to the MEGA 7.0 software [18]. To generate a phylogenetic tree, alignments were edited in the MEGA 7.0 software. Bootstrap analysis was performed on 1000 generations, and the phylogenetic tree was constructed using the neighbor-joining method.

### 2.4. Histopathology Sample Preparation

For histopathology examination, skin tissue with red mark lesions were immersed in 10% neutralized buffered formalin. Fixed skin tissues were trimmed and dehydrated using ethanol and then embedded in paraffin blocks. The paraffin blocks were sectioned, and the sections were stained with hematoxylin and eosin, examined by light microscopy, and digitally scanned by Xenos, Inc. (Seoul, Korea).

### 2.5. Morphological Analysis

Red spot skin lesion and spleen samples immersed in 2.5% glutaraldehyde solution were used for analysis. Samples were maintained at 4 °C overnight. Then the fixed samples were washed using PBS (0.1 M, pH 7.2) and post-fixed for 1 h in 1% osmium tetroxide (Sigma-Aldrich, MO, USA) at room temperature. The samples were then washed with PBS, dehydrated in a graded sequence of 50%, 60%, 70%, 80%, 90%, and 95% alcohol for 10 min, dehydrated twice in 99.9% ethanol for 1 h, and then substituted with propylene oxide (Sigma-Aldrich MO, USA). Infiltration was conducted using diluted epoxy resin (Sigma-Aldrich) mixed with PBS. The samples were embedded in epoxy resin for 1 h and then polymerized overnight at 80 °C. Serial sections were obtained by programmable microtome (Reichert-Jung 2050, ALT products, MA, USA) and the semithin sections were then stained with toluidine blue and examined by light microscopy to select areas with severe inflammation for use in TEM analysis. The selected areas were cut into thin sections using an ultramicrotome (MT-XL, RMC products, MA, USA) and then collected on a copper grid. The ultrathin sections were cut, stained with saturated 4% uranyl acetate (Sigma-Aldrich, MO, USA), and 4% lead citrate (Sigma-Aldrich), and then observed using a transmission electron microscope operating at 80 KV (JEM 1010, Shiga, Japan).

## 3. Results and Discussion

### 3.1. Sample Examination

Examination of exterior lesions showed negative results for any parasitic and fungal infections, as well as no particular signs of viral and bacterial infections. The results of post-mortem bacterial detection using PCR showed a negative result on *F. psychrophilum* [19] and any other viral diseases, such as infectious hematopoietic necrosis, infectious pancreatic necrosis, and viral hemorrhagic septicemia, which are known as major diseases in the rainbow trout industry [20]. The collected kidney, spleen, and liver also tested negative for fish pathogenic bacteria, such as *Aeromonas* sp., *Flavobacterium* sp., and *Pseudomonas* sp. [12]. Therefore, the cause of the studied disease had not been clearly established. However, RLO, which is described to be related to the RMS outbreak, was the only pathogens detected in our study.

### 3.2. Detection of Rickettsia and Phylogenetic Analysis

**Figure 2:**
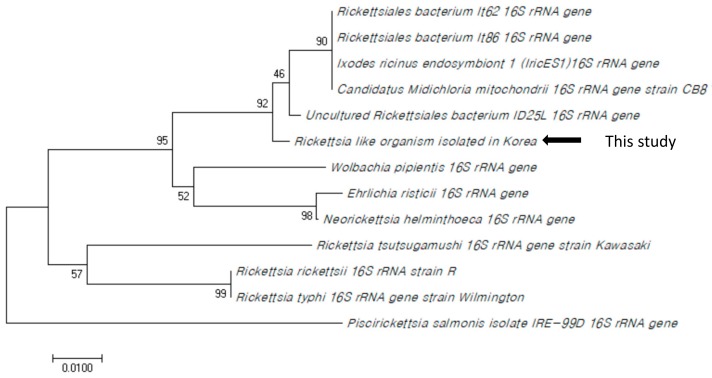
Phylogenetic analysis using partial sequence of the *Rickettsia*-like organism (RLO) 16S rRNA gene (188bp). The tree was constructed using the neighbor-joining method on MEGA 7.0 with a bootstrap of 1000 generations (GenBank accession number: HG793392.1, AF037211.1, AJ880275.1, U12457.1, AY498637.1, L36217.1, D38625.1, NR_118679.1, EU555284.1, AF525481.1, AF525482.1, AF179630.1. This study: MK 968141).

Some studies have shown that RLO can be found in both the spleen and skin using PCR. However, in our study, a positive result was only shown by the skin lesions [21] and all five samples collected for the PCR assay showed a positive result. However, the spleen, liver, and kidney samples showed negative results. A phylogenetic tree was constructed using the partial sequence of the 16s rRNA gene of the organism, and the result suggested that the pathogen was similar to the RLO isolated from a lake in southern Idaho, USA, which was responsible for RMS in rainbow trout fisheries in USA [7] (Figure 2).

The similarity value between strains were calculated, and the RLO isolate in Korea was similar to strain ID25L in 99.5% and IT86 and IT62 in 98.6%. To confirm the correlation between RLO and the disease, we performed PCR analysis of the skin and organs of the five rainbow trout that did not show the red mark but raised in the same water tank, and the result was negative. This result provided an explanation for the cause of the disease.

### 3.3. Histopathology Analysis

**Figure 3:**
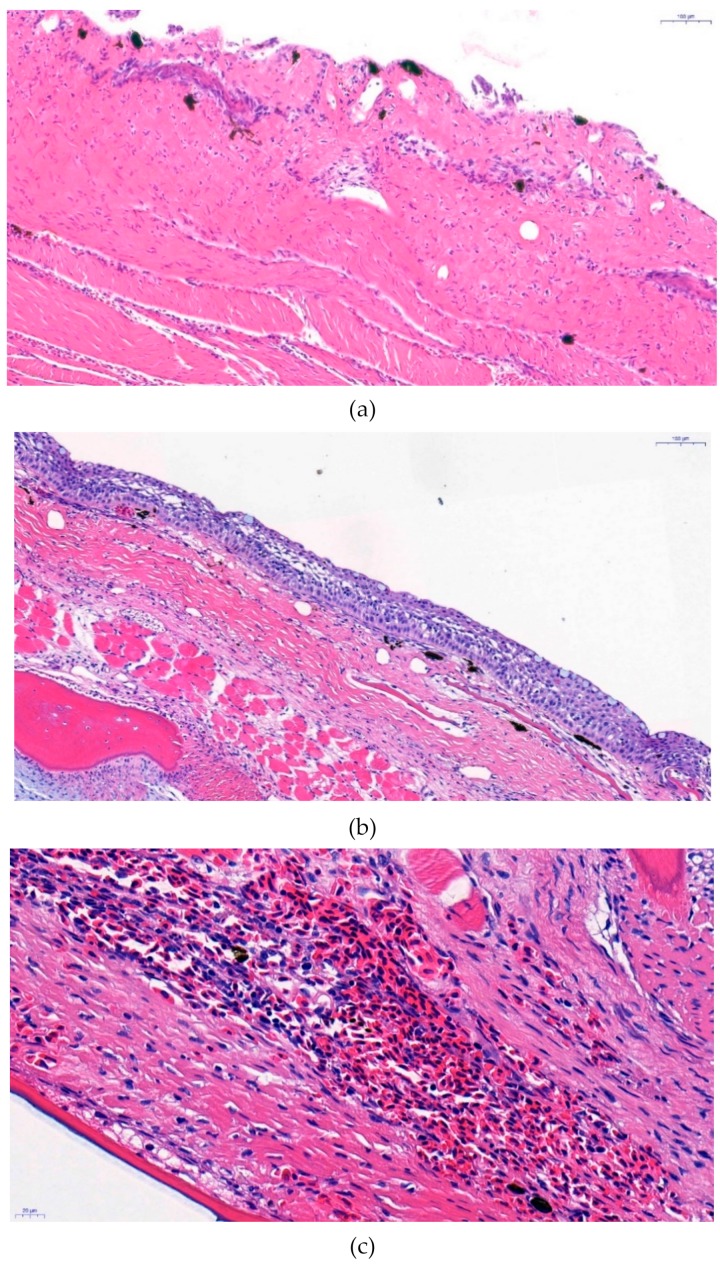

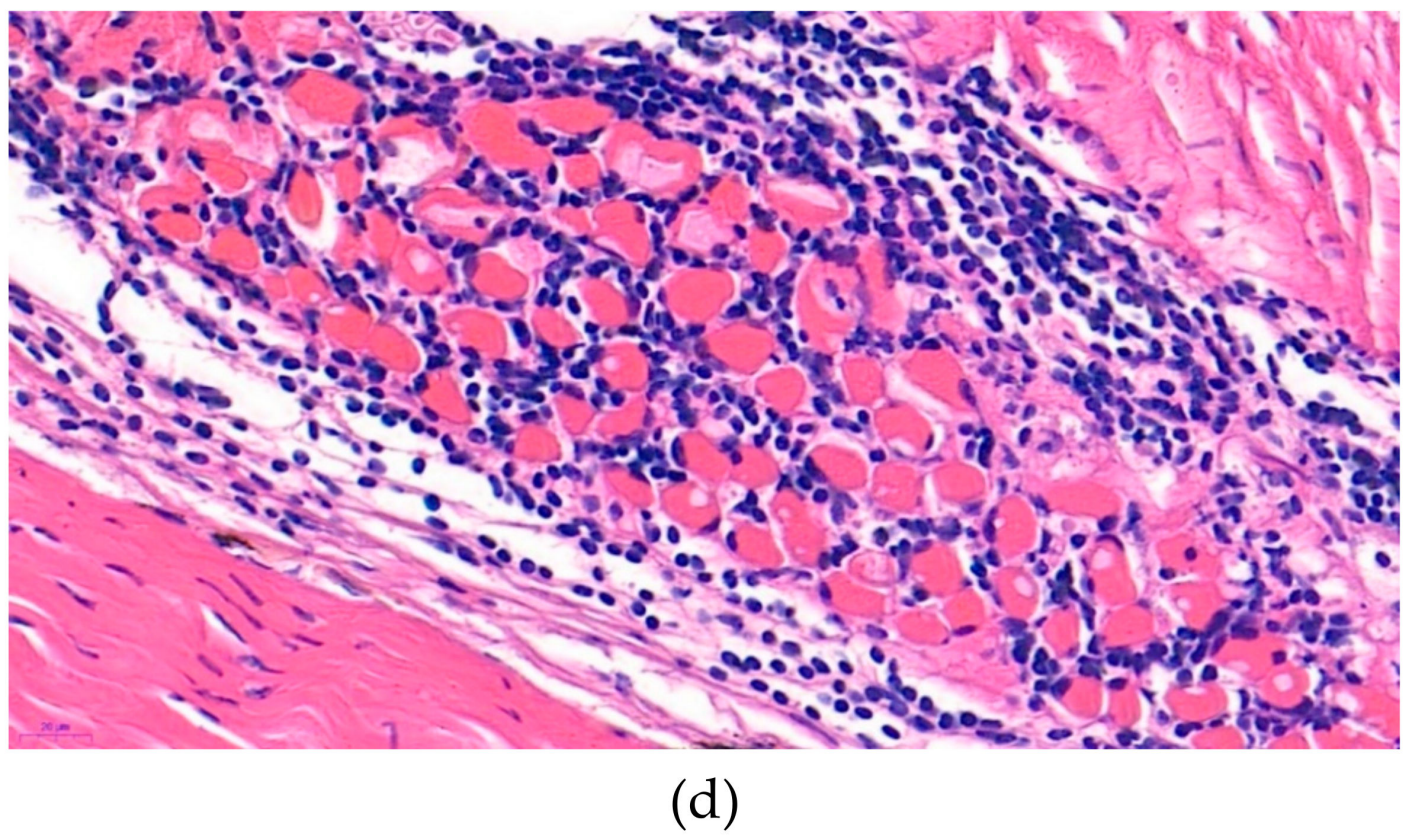
Histopathological analysis of hematoxylin and eosin-stained sections observed under a light microscope (400×). (**a**) Rickettsial infection-induced inflammatory lesions in the stratum spongiosum area, which was heavily thickened and showed scale disruption. (**b**) Histology of fish not showing the clinical symptom and having normal scale and stratum spongiosum, which was thinner compared to that in Figure 3 (**a**). (**c**) Congestion of erythrocytes in the outer layer of the dermis with macrophage and mononuclear cell infiltration. (**d**). Infiltration of several mononuclear cells, including lymphocytes, due to inflammation along the muscle tissue.

Previous studies have reported extensive inflammation in the dermis and stratum spongiosum areas, which involve all the layers of the epidermis to subcutis of fish [22]. In our study, we detected a highly thickened layer of the stratum spongiosum of the affected fish. This part of the skin showed signs of an inflammatory reaction, such as infiltration of lymphocytes, monocytes, and macrophages, compared to the opposite area of the skin that was not yet affected by the microorganism. The outermost layer of the skin was sloughed with a disruption of the scales due to severe swelling and inflammation. Thus, a hemorrhage was observed on the skin surface with mononuclear cell infiltration. Furthermore, the underlying muscular tissue that commonly showed moderate congestion of erythrocytes and cellular infiltration of immunocytes, such as lymphocytes, presented histological lesions that indicate an RMS infection [1] (Figure 3).

Since all these signs are the most common signs of RMS in rainbow trout, the disease investigated in this study could be easily diagnosed as RMS. However, in histopathology analysis, the spleen, liver, and kidney showed no signs of infection.

### 3.4. TEM Analysis

**Figure 4:**
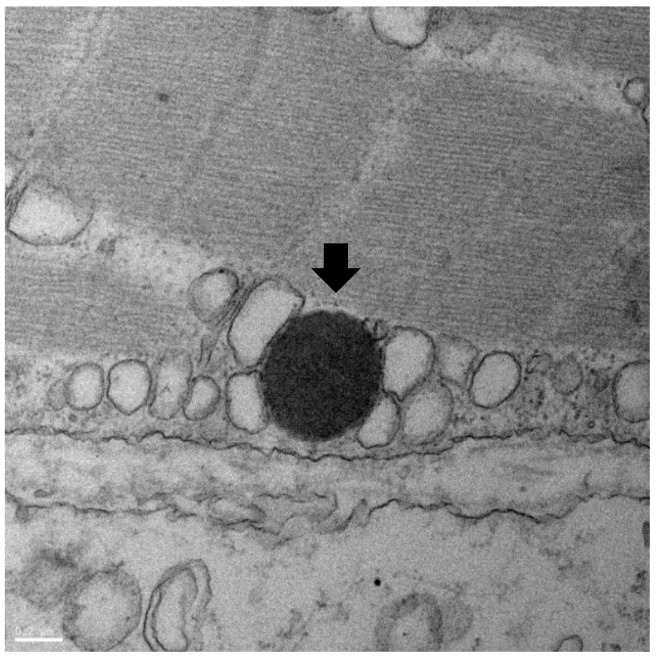
RLOs detected in the transmission electron microscopy (TEM) assay: Microorganisms with a diameter of 500 nm and a trilaminar layer.

Using TEM, hyper-pigmented, round-shaped microorganisms were detected in five skin samples of the affected fish. The diameter of the organism observed ranged from 200 to 500 nm, and, due to its oval shape, the length of the microorganism varied on the cross-sectioned area (Figure 4). Since the maximum length was measured as 500 nm, we estimated the size of this microorganism as approximately 500 nm. In addition, the microorganism had a clear outer membrane indicating a trilaminar layer, which is a representative characteristic of RLO [11]. The organisms were distributed in the area between muscle cells and around nerve bundles.

### 3.5. RMS Infection in Rainbow Trout Fry

The spleens collected from the affected rainbow trout were also used for TEM analysis, following prior studies, but no RLOs were detected in the erythrocytes or macrophages of the spleen samples [10]. However, because the result of PCR analysis was negative, this can give an explanation for the negative result in the TEM assay. The reason why RMS remains a syndrome is that the cause of the disease has not been clarified due to difficulties in culturing *Rickettsia* [23]. Moreover, in previous studies, PCR and TEM analysis results showed that RLO was not detected in every fish showing RMS signs [11]. However, in our study, the same microorganism was detected in every individual showing the clinical signs, but not in the fish that did not show the signs. Thus, the cause of the disease can be speculated. There are many reports of RMS in various countries, and economic losses are the main problems with RMS because this disease renders market-sized rainbow trout inappropriate for sale. However, in our study, we noticed that the disease only occurred in rainbow trout fry weighing less than 30 g, and not in rainbow trout that weighed more than that. Even though the facility used both open and closed recirculating aquaculture systems, the disease only occurred in rainbow trout fry. Therefore, horizontal transmission of the disease may vary on immune status and conditions of individuals and further study about the consequence seems to be needed. Another interesting finding of our study was that the affected rainbow trout fry showed signs of loss of appetite and all died in the end, and we could not observe similar signs in any live fish growing in the same water tank. The pathogenicity of this microorganism is not usually discussed because it was not related to the mortality of fish in prior studies [1]. However, in our study, we can speculate that this RLO may be an opportunistic pathogen that can cause secondary infection in rainbow trout fry or can affect the fish immune system under poor environmental and stressful conditions. This might have occurred because the immune response for Rickettsial infection in fry can be weaker compared to adult-sized ones. Since the examination results of viral, bacterial, and fungal infection in the affected fish were negative, the pathogenicity of the RLO and its influence on the immune status and growth of fry should be considered in further studies.

Taken together, this study was the first report of RMS in a rainbow trout farm in Korea. Due to warm weather and water temperature, there are not many reports of the disease in Asian countries. The size of rainbow trout fisheries is relatively small in Korea, compared to that in other countries, and this may be a reason why there were no report of this disease until now. Considering that the market of rainbow trout is rapidly increasing and an RMS outbreak is related to the mortality of rainbow trout fry, we believe investigations of the treatment and prevention of this disease are urgently needed in Korea, especially because there are not many reports of RMS in rainbow trout fry farmed in other countries. Moreover, the correlation of the microorganism and the immune system may be different between rainbow trout fry and adult rainbow trout. Therefore, the pathogenic role of the microorganism in the host must be further studied, and appropriate control measures should be established to prevent this disease.
